# Effect of Electrode Type on Electrospun Membrane Morphology Using Low-Concentration PVA Solutions

**DOI:** 10.3390/membranes12060609

**Published:** 2022-06-11

**Authors:** Zane Zelca, Andres Krumme, Silvija Kukle, Mihkel Viirsalu, Laimdota Vilcena

**Affiliations:** 1 Institute of Design Technology, Faculty of Materials Science and Applied Chemistry, Riga Technical University, LV-1048 Riga, Latvia; silvija.kukle@rtu.lv (S.K.); laimdota.vilcena@rtu.lv (L.V.); 2 Laboratory of Polymers and Textile Technology, Department of Materials and Environmental Technology, Tallinn University of Technology, 19086 Tallinn, Estonia; andres.krumme@taltech.ee (A.K.); mihkel.viirsalu@taltech.ee (M.V.)

**Keywords:** electrospinning, nano- and microfibers, polyvinyl alcohol, electrode type, molecular weight

## Abstract

Electrospun polymer nanofiber materials have been studied as basic materials for various applications. Depending on the intended use of the fibers, their morphology can be adjusted by changing the technological parameters, the properties of the spinning solutions, and the combinations of composition. The aim of the research was to evaluate the effect of electrode type, spinning parameters, polymer molecular weight, and solution concentration on membranes morphology. The main priority was to obtain the smallest possible fiber diameters and homogeneous electrospun membranes. As a result, five electrode types were selected, the lowest PVA solution concentration for stable spinning process was detected, spinning parameters for homogenous fibers were obtained, and the morphology of electrospun fiber membranes was analyzed. Viscosity, conductivity, pH, and density were evaluated for PVA polymers with five different molecular weights (30–145 kDa) and three concentration solutions (6, 8, and 10 wt.%). The membrane defects and fiber diameters were compared as a function of molecular weight and electrode type. The minimum concentration of PVA in the solution allowed more additives to be added to the solution, resulting in thinner diameters and a higher concentration of the additive in the membranes. The molecular weight, concentration, and electrode significantly affected the fiber diameters and the overall quality of the membrane.

## 1. Introduction

A large amount of research has been published to gain an understanding of the electrospinning process and its control, resulting in the opportunity to obtain the desired fiber diameters and morphology. There have been widespread attempts to improve the quality of fiber webs to expand their applications. Therefore, innovations such as coaxial electrospinning, mixing and multiple electrospinning, core–shell electrospinning, blow-assisted electrospinning, and bubble electrospinning have been proposed [1,2,3]. Several technologies have been combined with electrospinning to produce materials, e.g., electrospinning and electrode printing [4], electrospinning and electrospraying [5], and other techniques [6]. Unlike other membrane production methods (for instance, the non-solvent-induced phase separation process and solution phase inversion method), electrospinning does not require additional solvents, the fiber diameters and porosity are relatively easy to control, and the membrane consists entirely of fibers (not only porous but also completely fibrous) with high surface area-to-volume or length-to-diameter ratios [7,8].

Most published studies focused on the morphology of PVA electrospinning fibers depending on the intended use in a particular field, the additives used, or some specific technical parameters, but there have not been many studies that focused directly on the effect of different electrodes on PVA fiber web morphology. This can be explained by the limited technical possibilities within institutions, whereby there are not many laboratories equipped with electrospinning equipment, in which several electrodes of different shapes can be used. Therefore, three different electrospinning devices were used in this study (in Estonia and Latvia), with a total of five different electrodes. Electrospinning was performed using the same solutions and parameters, comparing the qualitative morphology, diameter, and productivity.

The equipment used can affect the final result quality and productivity, in terms of electrode shape, surface area, direction of fiber spinning, distance between electrodes, collector movement and shape [9], voltage, flow rate, and other specific parameters depending on the equipment.

Different approaches to scale up production have been explored by increasing the number of electrodes using multiple needles or free-surface technologies, as well as hybrid methods. Needleless and centrifugal methods have gained the most attention. In some cases, the production rate reached 450 g/h [10]. The electrodes used by mass electrospinning methods include multi-needle and needleless (free-surface) technologies (rotating cylinder and wires, single wire, hollow porous tube, and others) [11,12]. The electrospinning method utilizing a rotating spinneret is very effective, with cylinder, disc, ball, and wire electrodes being the most commonly reported [12,13].

If the properties of the fibers are compared between vertical and horizontal electrospinning systems, gravity can affect the electrospinning process. The shaft type spinning system had the widest size distribution of fiber diameter, while the converse type had the narrowest size distribution; but the size distribution of the horizontal type spinning system was intermediate [14].

The choice of electrode affects not only the productivity but also the parameters of the fibers obtained, such as fiber diameter, porosity, and web quality. For example, when comparing needle-type and single-wire machines, fibers with 80% drug additives were 10% thinner than needle-spun fibers [15].

When using a circular electrode collector, fiber membranes showed 1.56-fold higher productivity and uniformity compared to fiber membranes formed via the traditional electrospinning process using a flat collector [9].

In addition, parameters such as humidity and temperature are essential for solvent evaporation. The choice of solvent significantly affects the structure of the membrane. By combining different solvents, it is possible not only to control the evaporation process during electrospinning, but also to obtain, for example, nanofibers with a porous surface [16,17].

Even a minor parameter such as the fiber support material has an important effect on the fiber diameter. Polypropylene nonwoven material provided the lowest fiber diameter compared to black paper, various woven and knitted fabrics, carbon weaving, and aluminum foil surface using the roller electrospinning method [18].

Electrospun fiber membranes have a wide range of applications, which are still expanding. For example, the electrospun fibrous structure can be used for wound healing, absorbable dressings and topical preparations, grafts, stents, sclerotherapy balloons, and diagnostic sensors [19,20], as the carriers of microorganisms, stem cells, proteins, and nucleic acids in therapeutic applications [21], in filtration and thermal insulation, and in the manufacture of protective clothing, sensors, and conducting devices [22].

Polyvinyl alcohol (PVA) is widely used in electrospinning using different molecular weights and concentrations, but the effect of different electrodes on fiber morphology and diameter has not been extensively studied. The choice of electrode affects not only the fiber quality but also the overall productivity, which is important when using low-concentration solutions. Determining the lowest concentration of PVA solution for each electrode is an important reference point for predicting the quality of the membranes, the amount of additive, the spinning stability, and the smallest possible diameter to be obtained. The quality and efficiency of nanofibers are generally based on the fiber diameter and membrane sample structure [23].

In order to obtain the smallest possible fiber diameters from low-concentration solutions, many factors must be taken into account. Using too low of a solution concentration and polymer molecular weight will result in membrane defects, film areas, or no fiber formation. Therefore, it is important to control the viscosity, electrical conductivity, and surface tension of the solution. A lower-concentration solution results in the formation of more beads, facilitating the determination of the solution viscosity limits in micrographs [24].

The quality of fiber web morphology is essential in practical membrane applications such as filter materials, gas barriers, materials for microbiology and medicine, energy storage, sensors, and smart textiles, where porosity, low density, thin coatings, and a high surface area-to-volume ratio are important. By changing the electrospinning parameters, it is possible to change not only the morphology (the overall quality of the membrane) and shape of the fibers, but also the permeability (porosity), thickness, and final amount of material obtained [21,22,23,24].The aim of this research was to evaluate the effect of solution parameters (such as polymer molecular weight and solution concentration) and equipment parameters (such as electrode type and distance between electrodes) on fiber membranes to achieve the lowest possible fiber diameters. As a result, spinning solutions with three concentrations (6, 8, and 10 wt.%) and five different molecular weights were evaluated (30–145 kDa), and they were spun using five different electrodes (fixed single wire, rotating five wires, rotating cylinder, needle, and pike) and two different distances between the electrodes (15 and 18 cm).

## 2. Materials and Methods

### 2.1. Materials

The polyvinyl alcohol polymer matrix used in this research was obtained from Sigma-Aldrich Chemical Company (Darmstadt, Germany). Series of experiments were performed using samples with different PVA molecular weights: 30–70 kDa (degree of hydrolysis 87.0–90.0, melting point 250 °C), 89–98 kDa (degree of hydrolysis 99.0, melting point 250 °C), 125 kDa (Mowiol 20–98, degree of hydrolysis 98.0–98.8, melting point 200 °C), 130 kDa (polyvinyl alcohol 18–88, degree of hydrolysis 86.7–88.7, melting point 200 °C), and 145 kDa (Mowiol 28–99, melting point 230 °C). For each PVA molecular weight, three polymer concentrations in an aqueous solution for electrospinning were obtained (6, 8, and 10 wt.%). Laboratory distilled water (conductivity 1 µS/cm) was used to prepare the PVA solutions.

### 2.2. Characterizations

To assess the suitability of the prepared solutions for electrospinning, the density (aerometer), conductivity (conductometer Greisinger GLF-100, Regenstauf, Germany), viscosity (Viscometer Viscolead adv, Fungilab S.A., 100 rpm, used tip L6, Barcelona, Spain), and pH (pH/ORT Tester AD14, ADWA Instruments, Bucarest, Hungary) of each solution were determined at 20 ± 0.15 °C. 

Spinning process stability was evaluated according to three levels—good, fibers formed throughout spinning time; average, fibers were consistently not formed throughout spinning time; bad, only a few fibers were obtained, with practically no electrospinning or electrospraying. Cases where spinning did not occur were marked by an X.

Fiber quality and diameters were analyzed from the micrographs obtained using a. optical microscope (Zeiss Axioskop-2) and scanning electron microscope (SEM Mira Tescan, Bern, Czech Republic); samples were coated with gold (5–20 nm) before the observation. The image analysis program ImageJ was used for diameter measurements (at least one hundred measurements at five different locations for each sample).

### 2.3. Solution Preparation for Electrospinning

Twelve electrospinning solutions were obtained according to the information in Table 1. The solutions were designated by two numbers; the first was the PVA wt.% in solution, and the second was the molecular weight.

### 2.4. Fabrication of Membranes

Various electrode types (cylinder, pike, five rotating wires, single fixed wire, and needle) were used to obtain membranes by electrospinning using equipment (Nanospider Lab 200 (equipped with rotating cylinders/wires or a fixed pike electrode); Nanospider NS LAB (equipped with a single fixed wire)) from Elmarco, while a 5 mL syringe with a flow control pump (Yflow SD) was used for needle-type spinning (see Table 2 and Table 3). An electrospinning voltage of 10–67 kV was used depending on the electrode type and distance between electrodes, which was adjusted according to the molecular weight of each polymer and the concentration of PVA in the solution (Table 2). The distance between the electrodes was 15 and 18 cm, the electrode rotation speed was 4 rpm (for the five-wire and cylinder-type electrodes), and the collector rotating speed is 300 rpm (for the needle-type electrode). Electrospinning took place at 21–25 °C with the relative air humidity ranging from 22% to 28%. The needle feeding rate was 0.3 mL/h. The fixed-wire electrode was coated with a solution using a carriage speed of 30 mm/s and a 0.6 mm nozzle (see Table 3). The dimensions of the electrodes are shown in Table 3. Polypropylene nonwoven material was used as a support material.

## 3. Results and Discussion

### 3.1. PVA Solution Characteristics

**Figure 1:**
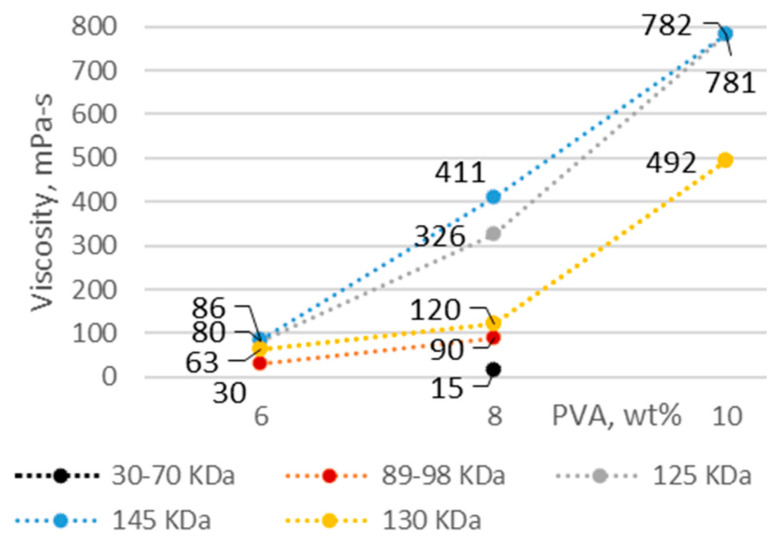
The viscosity parameters of spinning solutions depending on the concentration and molecular weight of polyvinyl alcohol.

**Figure 2:**
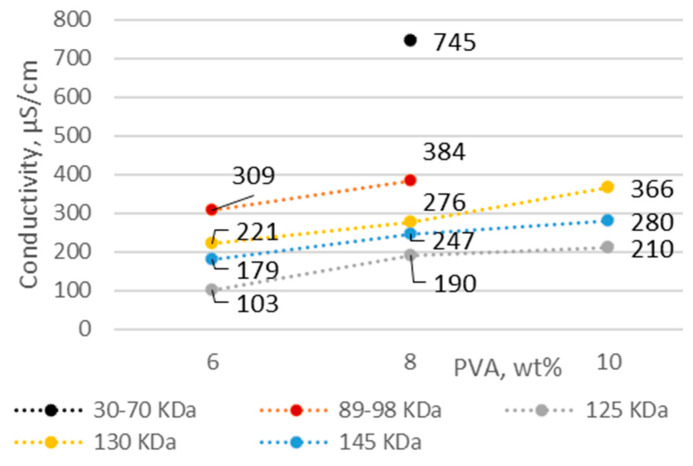
The electrical conductivity values of electrospinning solutions depending on the concentration and molecular weight of polyvinyl alcohol.

**Figure 3:**
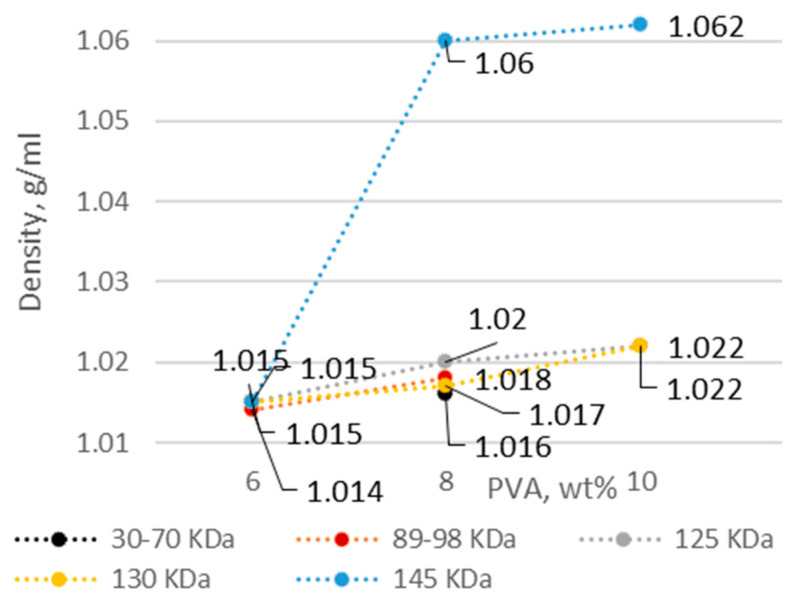
The density values of electrospinning solutions depending on the concentration and molecular weight of polyvinyl alcohol.

**Figure 4:**
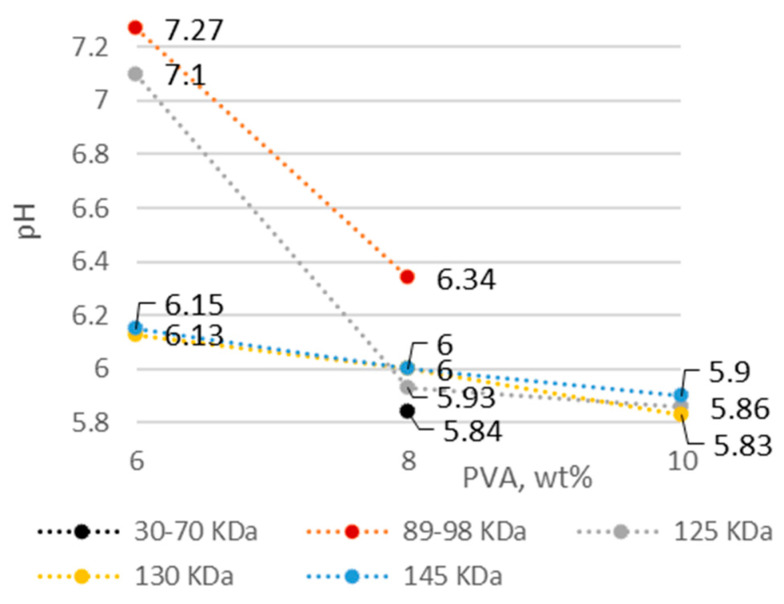
The pH values of electrospinning solutions depending on the concentration and molecular weight of polyvinyl alcohol.

Polyvinyl alcohol was used as the fiber base material. The solution concentration and molecular weight of PVA are important factors mediating the solution properties, thus affecting the quality of the fiber webs. The percentage of PVA with the lowest concentration in solution was selected on the basis of previous studies [25,26,27]. As the concentration and molecular weight of PVA increased, the electrical conductivity, viscosity, and density increased, while the pH became more acidic (Figure 1, Figure 2, Figure 3 and Figure 4).

The solution viscosity increased with increasing PVA concentration according to a power-law relationship. As shown in Figure 1, the viscosity of sample 8PVA30_70 was 15 mPa·s, while that of sample 6PVA89_98 was 30 mPa·s, both of which were insufficient to obtain fibers. Sample 8PVA89_98 with a viscosity of 90 mPa·s and solutions of all three PVA concentrations with molecular weights ranging from 125 to 145 kDa were suitable for electrospinning. The highest viscosity was obtained for samples with 10 wt.% PVA, which can be used for solutions where additives with low viscosity are needed. Solutions with 6 wt.% PVA had the lowest viscosity and the lowest spinning stability (average or bad) using cylinder-, wire-, and pike-type electrodes (Table 4).

The electrical conductivity of the solvent was low (1 µS/cm); it can be seen that increasing the concentration of PVA increased the electrical conductivity of the solution. Electrical conductivity was affected not only by the concentration of PVA but also by the molecular weight and degree of hydrolysis. All solutions analyzed had sufficient electrical conductivity to obtain fibers (Figure 2). The voltage required for electrospinning was not correlated with the electrical conductivity of the solutions (Table 2).

The density of the solutions was affected by both PVA concentration and molecular weight, increasing more quickly higher-molecular-weight solutions. The pH of the solutions became more acidic with decreasing water content and increasing PVA concentration (from 6 wt.% to 10 wt.% PVA), whereas no significant effect of molecular weight was observed (Figure 3 and Figure 4). A pH near 2 was previously found to significantly affect the fiber size [28], but no values obtained in this study (5.8–7.2) reached this limit.

Taking into account the properties of the solutions, polymers with a molecular weight of 125–145 KDa were selected for further research, and particular attention was paid to the lowest concentration of 6 wt.%. As shown in Table 4, solutions of all concentrations could be electrospun (except for sample 6PVA145 electrospun with wires). It can be seen that using a higher-molecular-weight polymer allowed obtaining a more unstable spinning process at a concentration of 6 wt.% using rotating wire- and cylinder-type electrodes (Table 4). The same electrode rotation speed (4 rpm) was used for all concentrations, resulting in a more unstable spinning process at lower PVA concentrations.

**Figure 5:**
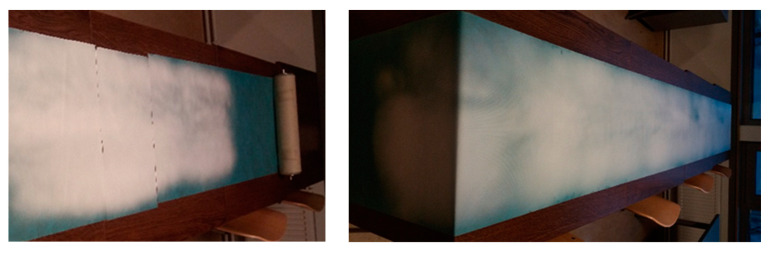
Result obtained after spinning on the support material for 1 h at a speed of 0.003 m/s using a cylinder-type electrode: sample 10PVA130 (**left**) and 8PVA130 (**right**).

One study reported that PVA concentrations of 4–10 wt.% were used to obtain composites with additives that increase viscosity using a needle-type electrode [29]. Another study reported that uniform fibers were fabricated via syringe and foam electrospinning with higher fiber quality compared to the Nanospider using a droplet electrode (7–9 wt.% PVA solution concentration), while a more homogeneous membrane was obtained with syringe compared to foam electrospinning (5 wt.% PVA concentration) [30]. As can be seen (Table 4 and Figure 1), the concentration of PVA and the chosen electrode had an effect on the spinning process stability and quality of the fiber membrane due to the viscosity (lowest quality for PVA with a molecular weight of 130 kDa; average and bad spinnability when using pike, rotating wires, and cylinder). Although the spinning process was stable, the end result was more homogeneous when using a higher solution viscosity (8–10 wt.% PVA concentration) using 130 kDa molecular weight PVA and a cylinder-type electrode (Figure 5).

### 3.2. Effect of Electrode Type and Distance between Electrodes on Membrane Morphology

**Figure 6:**
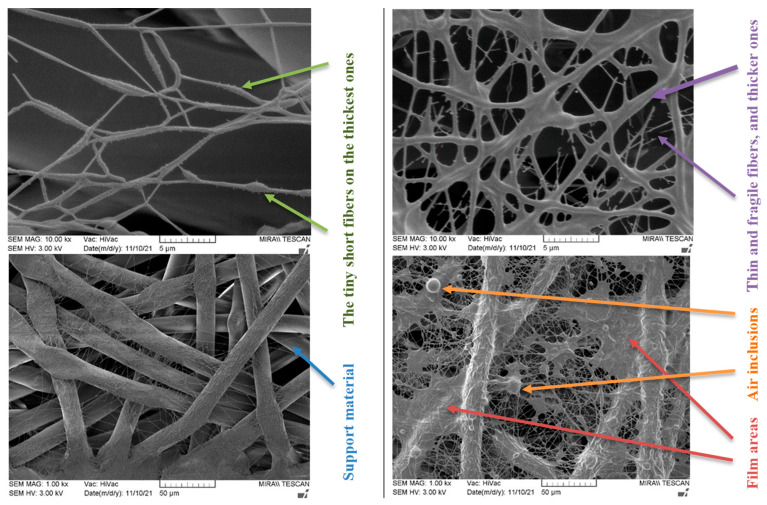
Electrospun membranes of sample 6PVA130 at an electrode distance of 150 mm using pike-type (**left side**) and needle-type (**right side**) electrodes.

The 6 wt.% PVA solution with the least suitable viscosity for spinning (i.e., sample 6PVA130 with a viscosity of 63 mPa·s; Figure 1) was spun using different electrodes at the same electrode distance; the fiber quality was evaluated by SEM micrographs. As shown in Figure 6, the pike-type vertical electrospinning device produced higher-quality fibers compared to the needle-type electrode, with the vertical placement leading to an overall more homogeneous web quality. The pike-type electrode produced fibers that did not form distinct diameters, while an uneven relief on the surface of the fibers could also be seen (ranging from tiny short fibers to thick ones; Figure 6, left).

When the viscosity of the solution was increased to its optimum range, the morphology of the nanofibers changed from beaded (see Supplementary Materials Figure S1) to a spindle shape. Once the viscosity reaches its optimal range, viscoelastic forces prevent the fragmentation of polymer chains, resulting in the continuous formation of uniform nanofibers [31].

On the other hand, needle-spun fibers showed an unstable spinning process, producing both thick, sticky fibers, which in some places also formed film areas and air inclusions, and very thin and fragile fibers, which were already broken in some places due to ventilation. Accordingly, the fiber membrane was very heterogeneous with many defects (Figure 6, right). Increasing the distance between the electrodes could prolong the evaporation time of water and improve the quality of the fibers, whereas a high feeding rate was another factor potentially leading to air bubble entrapment. Several beads are visible in the optical micrographs (Supplementary Materials Figure S1), indicating that the viscosity was too low, which could be solved by choosing a higher molecular weight.

**Figure 7:**
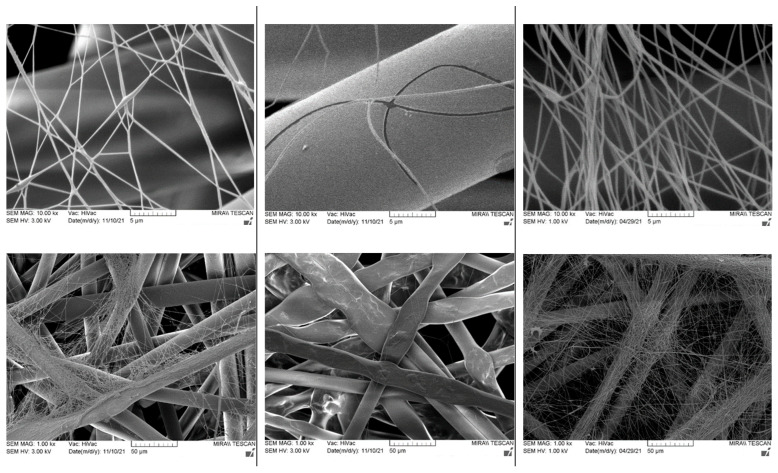
Electrospun membranes of sample 6PVA130 with an electrode distance of 180 mm using fixed-wire (**left**), five-rotating-wire (**center**), and rotating cylinder-type (**left**) electrodes.

Comparing the fiber membranes spun at distances of 15 cm and 18 cm between electrodes, the micrographs (Figure 6 and Figure 7) reveal that fibers with more uniform diameters were obtained when using a distance of 18 cm and a vertical spinning flow of the polymer. When the distance between electrodes was increased, the time for the solvent to evaporate increased, resulting in the collection of dry solid fibers on the target. On the support material, after 10 min of spinning, a different end result was obtained; most of the polymer was spun on the sample when using a needle-type electrode, but the fibers were defective. The largest number of high-quality and uniformly distributed fibers was obtained for the samples spun using cylinder-type and single-wire electrodes, whereas the sample spun with several rotating wires contained very few fibers.

The web of fibers obtained when spinning with one wire was homogeneous; the fibers tended to scatter around the fibers of the support material, and the diameters of the fibers were uneven throughout the length of the fiber, but no pronounced defects were observed (Figure 7, left). Beads were visible in the optical micrographs (Figure S1).

The web of fibers spun using several rotating wires was difficult to evaluate due to the small end result, but the diameters of the visible fibers were homogeneous (Figure 7, center). 

The diameters of the fibers spun using the cylinder-type electrode were seemingly uniform, while the overall membrane was homogeneous, with slight defects in the form of small film areas (Figure 7, right and Figure S2).

### 3.3. Membrane Morphology Depends on Concentration and Molecular Weight

Since the samples with the most homogeneous fiber diameters were obtained using a cylindrical electrode, the dependence of fiber diameter on the PVA concentration and molecular weight was further analyzed (Figure S2).

The 6 wt.% PVA solution with a molecular weight of 145 kDa formed a highly defective sample with more films than fibrous areas, while the 10 wt.% solution also formed film areas. Only the 8 wt.% PVA solution concentration allowed obtaining a homogeneous fiber membrane with good-quality fibers (Figure S2).

Solutions of all concentrations with a PVA molecular weight of 130 kDa yielded high-quality fibers with homogeneous diameters; the overall membrane quality was relatively homogeneous, while a droplet-like film could be seen in some areas due to the high voltage applied in the spinning process (Figure S2).

High-quality fiber membranes with homogeneous fiber diameters were also obtained when using 125 kDa PVA (6–10 wt.%) solutions (Figure S2).

As the viscosity and conductivity of the solution did not differ significantly between 125 kDa and 145 kDa solutions, it can be concluded that the concentration and molecular weight were the main influencing factors for the stability of the fiber spinning process and the quality of the membrane when using the same spinning parameters with a cylindrical electrode.

### 3.4. Fiber Diameter Evaluation

**Figure 8:**
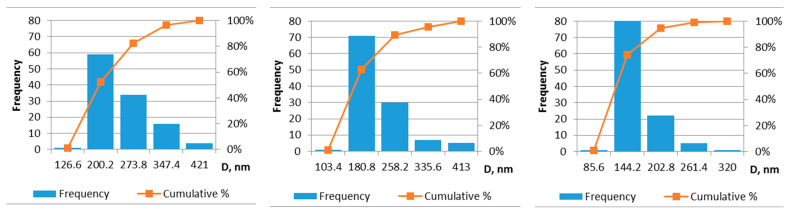
Histograms of electrospun fiber diameters: sample 6PVA125 electrospun using cylinder-type electrode (**left**); sample 6PVA130 electrospun using cylinder-type electrode (**center**); sample 6PVA130 electrospun using wire-type electrode (**right**).

As the solutions with molecular weights of 125 and 130 kDa had a high or very good membrane quality when using cylinder-type and single-wire electrodes, the distribution of fiber diameter at the lowest PVA concentration (6 wt.%) was analyzed (Figure 8).

The 6 wt.% PVA solutions spun using a cylindrical electrode produced different fiber diameters depending on the molecular weight. Several fibers tend to stick together; thus, the measurements were performed only for nonadherent fibers. The relative error of the measurements ranged from 4% to 5% (Figure S2 and Table 5).

The fiber diameters of sample 6PVA125 electrospun using the cylinder-type electrode ranged from 53 to 421 nm, with a mean of 201.8 ± 7.7 nm, whereas the fiber diameters of sample 6PVA130 electrospun using the cylinder-type electrode ranged from 26 to 413 nm, with a mean 168.4 ± 7.6 nm. As expected, the solution with lower viscosity produced fibers with a thinner diameter; the small difference in molecular weight also resulted in similar diameters. The fibers of sample 6PVA130 electrospun using the cylinder-type electrode featured a higher frequency of diameters up to 180 nm (Figure 8) compared to the membrane obtained using sample 6PVA125.

Comparing the membranes obtained from sample 6PVA130 electrospun using cylinder and wire-type electrodes, it can be seen that the wire-type electrode produced fibers with a smaller diameters (27 to 320 nm, with a mean of 117 ± 5.2 nm) and a more uniform diameter distribution (Figure 8, right).

## 4. Conclusions

Identifying PVA concentrations yielding the lowest-viscosity solutions is an important step in predicting the maximum amount of additives that can be added. Electrospinning polymer nanofiber membranes can be a useful strategy to transfer various additives via mass production. This study revealed that both viscosity and choice of electrode are very important for membrane quality in terms of morphology and productivity, while other electrospinning parameters must also be taken into account, although their comparison is hindered by the existence of different technical parameters. Fibers with the thinnest possible diameter and uniform distribution of diameters were produced by the single-wire electrode using a PVA solution with a molecular weight of 130 kDa and concentration of 6 wt.%. The 6 wt.% PVA solutions with a lower molecular weight resulted in viscosities too low for electrospinning (30–70 mPa·s). The cylinder-type and single-wire electrodes had a higher productivity compared to the five-wire electrodes when using the same parameters. The SEM micrographs evidenced beads and defects, indicating the low viscosity of the 6 wt.% 130 kDa PVA solution when electrospun using pike- and needle-type electrodes with a distance of 15 cm between the electrodes. The safest choice for obtaining high-quality fibers is the 8 wt.% PVA solution concentration using various electrodes and a wide range of PVA molecular weights. If the aim is to obtain fibers as thin as possible, the 6 wt.% PVA solution with a molecular weight of 130 kDa electrospun using a fixed wire-type electrode is the most appropriate choice, yielding fiber diameters in the range of 27 to 320 nm.

## Figures and Tables

**Table 1:** The time and temperature used for PVA solution preparation.

Sample	Molecular Weight, kDa	PVA Content in Solution, wt.%	Mixing Temp., °C	Stirring Time, h
8PVA30_70	30–70	8	10–120	3
6PVA89_98	89–98	6		
8PVA89_98		8		
6PVA125	125	6	90–100	2
8PVA125		8		
10PVA125		10		
6PVA130	130	6	75–90	
8PVA130		8		
10PVA130		10		
6PVA145	145	6	90–110	
8PVA145		8		5–13
10PVA145		10		

**Table 2:** The electrospinning parameters, electrode type and solution concentration used.

Electrode Type		Pike	Needle	Wire	5 Wires	Cylinder
Molecular Weight, kDa	PVA Solution Concentration, wt.%	Electrospinning Voltage, kV				
125 kDa	6	30			54	66
	8	26			49	66
	10	26			46	66
130 kDa	6	27	10	40	55	67
	8	27			57	66
	10	29	10	60	58	58
145 kDa	6	29			-	57
	8	29			49	57
	10	27			45	60

**Table 3:** Spinning systems depending on electrode type.

Pike	Needle	Wire	Rotating Wires	Cylinder
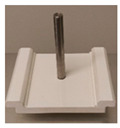	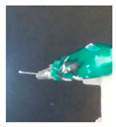	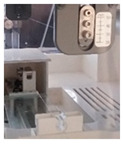	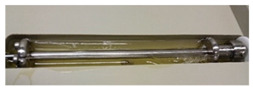	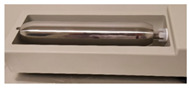
Converse type	Horizontal type	Converse type	Converse type	Converse type
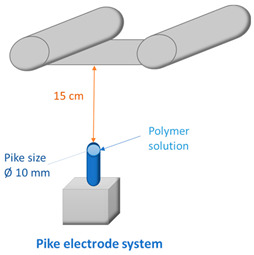			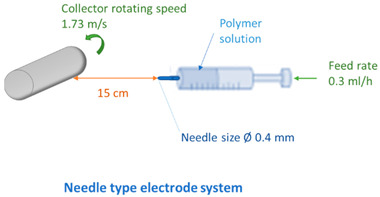	
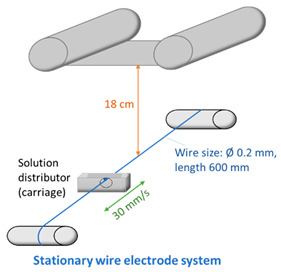 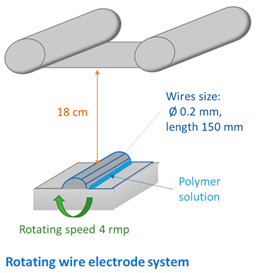 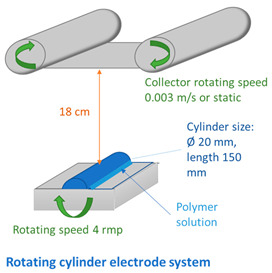				

**Table 4:** Spinning process stability.

Electrode Type		Pike	Needle	Wire	5 Wires	Cylinder
Molecular Weight, kDa	PVA Solution Concentration, wt.%	Electrospinning Voltage, kV				
125 kDa	6	G			A	A
	8	G			G	G
	10	G			G	G
130 kDa	6	A	G	G	B	B
	8	G			G	G
	10	G	G	G	G	G
145 kDa	6	G			X	G
	8	G			G	G
	10	G			G	G

Spinning process stability: G—good, A—average, B—bad, and X—did not spin.

**Table 5:** Statistical data of electrospun fiber diameters.

	6PVA125 (Cylinder)	6PVA130 (Cylinder)	6PVA130 (Wire)
Mean	201.8	168.4	117.0
−/+	7.7	7.6	5.2
Relative error	4%	5%	4%
Minimum	53	26	27
Maximum	421	413	320
Amplitude	368	387	293
Median	200	157	119
Mode	132	136	133
Standard deviation	75.3	73.9	50.6

## Data Availability

The data presented in this study are available in this article and supplementary materials.

## Supplementary Materials

The following supporting information can be downloaded at: https://www.mdpi.com/article/10.3390/membranes12060609/s1, Figure S1: The micrographs of the optical microscope, x500 and x100; Figure S2: Nanoweb morphology depend on concentration and molecular weight, electrospun with cylinder-type electrode.
