# Supplementary material for: Effect of Electrode Type on Electrospun Membrane Morphology Using Low-Concentration PVA Solutions
Source: Membranes (Basel). 2022 Jun 11;12(6):609. doi: 10.3390/membranes12060609 (PMC9229226; doi:10.3390/membranes12060609)
Supplement: Supplementary file 1 [file membranes-12-00609-s001.zip › membranes-1751214-supplementary.pdf]

Supplemented material

Effect of Electrode Type on Electrospun Membrane Morphol-ogy Using Low-Concentration PVA Solutions

Zane Zelca[a,*], Andres Krumme[b], Silvija Kukle[a] , Mihkel Viirsalu[b], Laimdota Vilcena[a]
[a]*Institute of Design technology, Faculty of Materials Science and Applied Chemistry, Riga Technical University, Latvia;*
[b] *Laboratory of Polymers and Textile Technology, Department of Materials and Environmental Technology, Tallinn University of Technology, Estonia.*
 * Corresponding author – Zane.Zelca@rtu.lv, Riga Technical University, Kipsala street 6, Riga, Latvia

Scanning electron microscope SEM Mira Tescan and optical microscope Zeiss Axioskop-2 used for S.1. and S.2. micrographs imaging. Electrospun fiber webs are spun from the solutions described in the table below.

PVA solutions preparation and designations

Sample	Molar weight, kDa	PVA content in solution, wt%	Mixing temp., °C	Stirring Time, h
6PVA125		6		
8PVA125	125	8	90-100	
10PVA125		10		2
6PVA130		6		
8PVA130	130	8	75-90	
10PVA130		10		
6PVA145		6		
8PVA145	145	8	90-110	5-13
10PVA145		10		

S.1. The micrographs of the optical microscope, x500 and x100

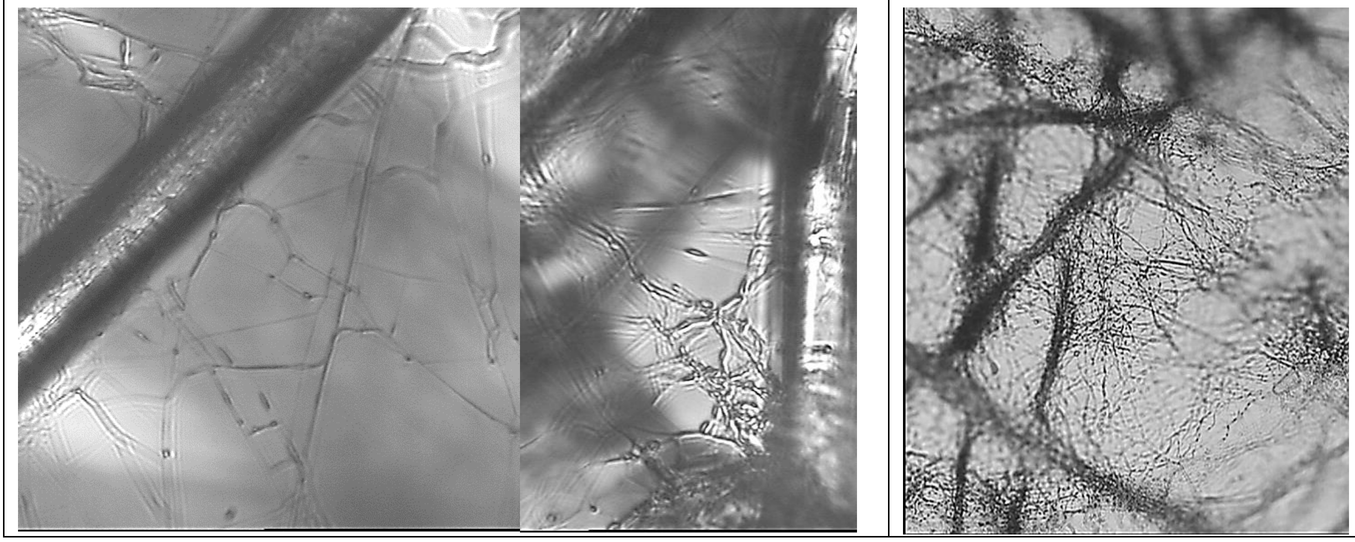

The solution 6PVA130 electrospun by: single wire electrode (left) and needle-type electrode (right)

S.2. Nanoweb morphology depend on concentration and molecular weight, electrospun with cylinder-type electrode

<table>
<tr><th>PVA</th><th>6 wt%</th><th>8 wt%</th><th>10 wt%</th></tr>
<tr><td rowspan="2">125.000 g/mol</td>
<td>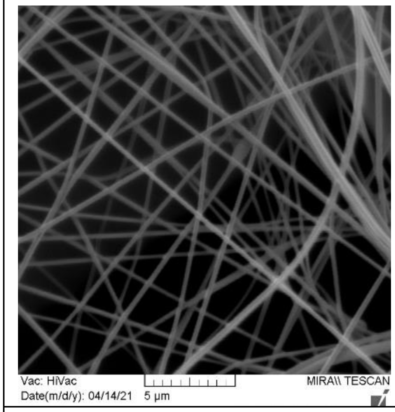
</td>
<td>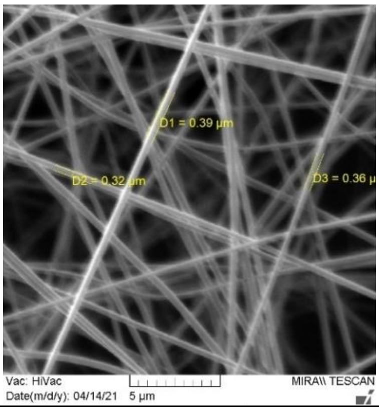
</td>
<td>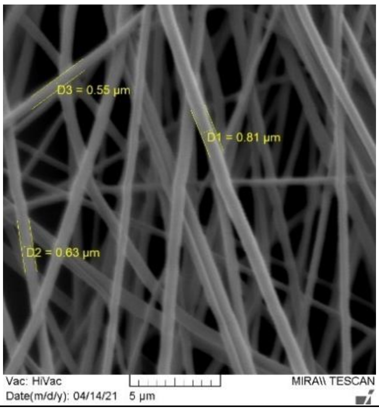
</td></tr>
<tr>
<td>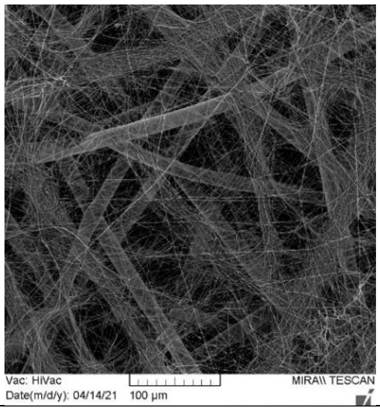
</td>
<td>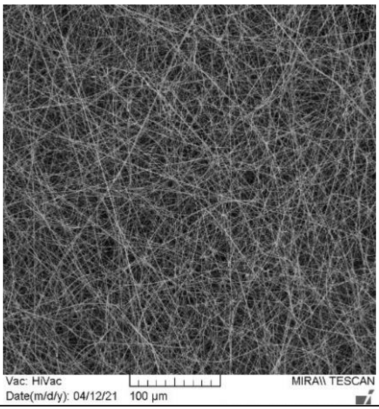
</td>
<td>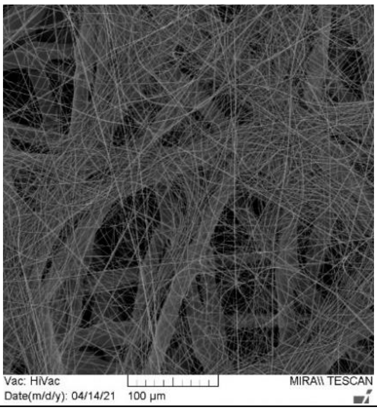
</td></tr>
<tr><td rowspan="2">130.000 g/mol</td>
<td>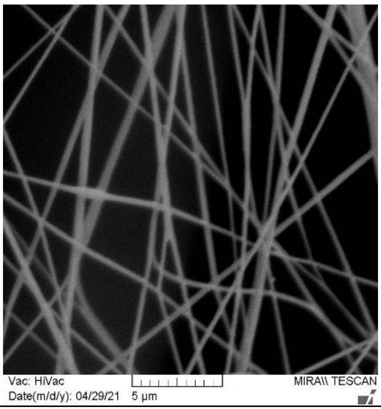
</td>
<td>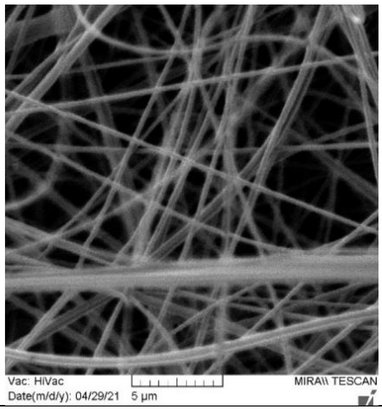
</td>
<td>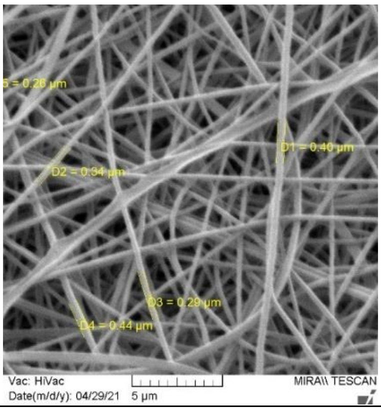
</td></tr>
<tr>
<td>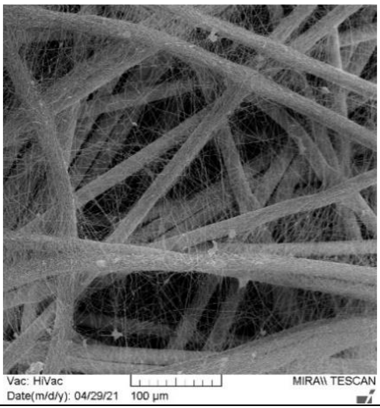
</td>
<td>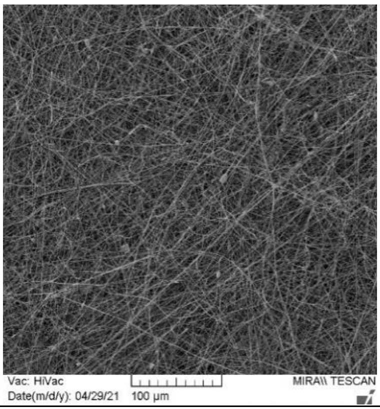
</td>
<td>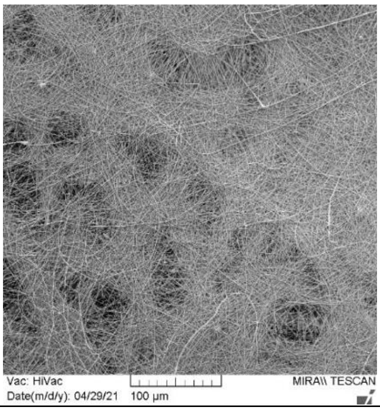
</td></tr>
</table>

<table>
<tr><th>PVA</th><th>6 wt%</th><th>8 wt%</th><th>10 wt%</th></tr>
<tr><td rowspan="2">145.000 g/mol</td><td>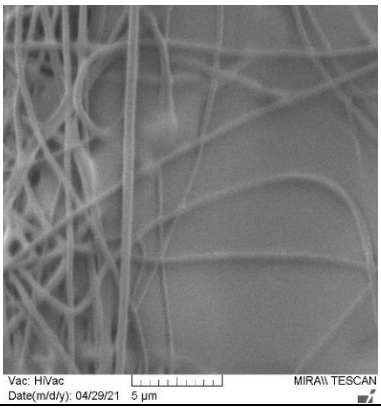
</td><td>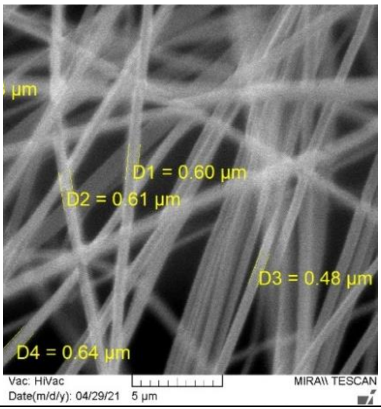
</td><td>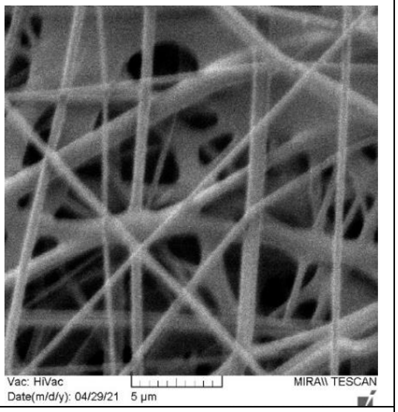
</td></tr>
<tr><td>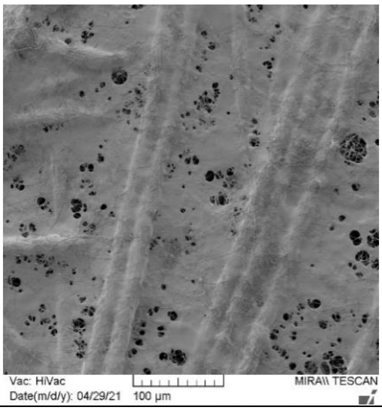
</td><td>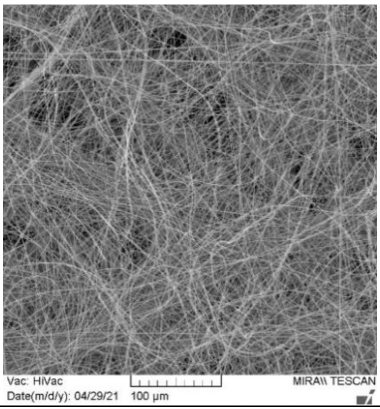
</td><td>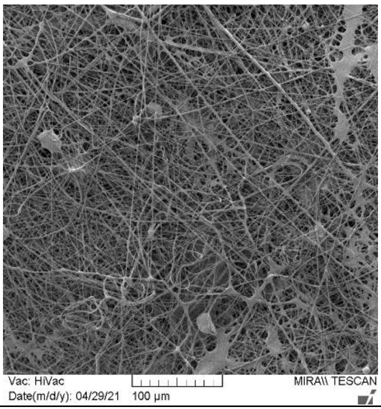
</td></tr>
</table>
